# Himalayan *Ficus palmata* L. Fruit Extract Showed In Vivo Central and Peripheral Analgesic Activity Involving COX-2 and Mu Opioid Receptors

**DOI:** 10.3390/plants10081685

**Published:** 2021-08-16

**Authors:** Devesh Tewari, Pawan Gupta, Sweta Bawari, Archana N. Sah, Davide Barreca, Maryam Khayatkashani, Hamid Reza Khayat Kashani

**Affiliations:** 1 Department of Pharmacognosy, School of Pharmaceutical Sciences, Lovely Professional University, Phagwara 144411, India; 2 Department of Pharmaceutical Sciences, Faculty of Technology, Bhimtal Campus, Kumaun University, Nainital 263136, India; sweta.bawari@sharda.ac.in (S.B.); archanansah@gmail.com (A.N.S.); 3 Shree S. K. Patel College of Pharmaceutical Education and Research, Ganpat University, Ganpat Vidyanagar, Mehsana 384012, India; pkg02@ganpatuniversity.ac.in; 4 Department of Pharmacology, School of Pharmacy, Sharda University, Knowledge Park III, Greater Noida 201310, India; 5 Department of Chemical, Biological, Pharmaceutical and Environmental Sciences, University of Messina, 98168 Messina, Italy; dbarreca@unime.it; 6 School of Iranian Traditional Medicine, Tehran University of Medical Sciences, Tehran 14155-6559, Iran; maryamkhaiiat@yahoo.com; 7 Department of Neurosurgery, Imam Hossein Hospital, Shahid Beheshti University of Medical Sciences, Tehran 1617763141, Iran

**Keywords:** *Ficus*, pain, hot plate, formalin, cyclooxygenase, neurogenic

## Abstract

Analgesic drugs like morphine and non-steroidal anti-inflammatory drugs exhibit several harmful effects. Here, we show for the first time the analgesic activity of *Ficus palmata* L. fruit extract (FPFE) on different analgesic rat models along with the in silico studies of some of the main phytochemicals of this plant. We performed in vivo pain models, along with in silico docking studies against the active site of COX-2 protein and mu-opioid receptors. A significant (*p* < 0.05) analgesic effect of FPFE was observed, and it was found that rutin has good pose and score as compared to diclofenac and morphinan antagonist (X-ligand), and psoralen has binding affinity almost equal to diclofenac, but a lower binding affinity as compared to rutin. The results proved that *F. palmata* fruits have the potential to ameliorate painful conditions.

## 1. Introduction

In clinical settings, the most widely used drugs for reduction of pain and inflammation are non-steroidal anti-inflammatory drugs (NSAIDs) [1]. Analgesic drugs like opiates and NSAIDs are not believed to be useful in all cases due to their low potency and adverse effects [2]. Both opiates and NSAIDs possess potential side effects like opiate poisoning, gastrointestinal disturbance and hepatic dysfunction [3]. This necessitates looking for beneficial alternatives. Medicinal plants can serve as a potential alternative source due to the presence of a diverse and complex variety of chemicals from which the discovery of novel analgesic agents is possible [4].

*Ficus palmata* Forssk. (Moraceae) is amongst one of the less explored species of *Ficus* genus found in the Himalayan region. Due to its abundance in the Himalayan region, the plant is also known as “Wild Himalayan fig”. In the local dialect of Kumaun region of India (Kumauni), the *Ficus palmata* figs are known as “Bedu”. These plants are of very high popularity in various traditions and have tremendous cultural importance in the western Himalayan region. *F. palmata* fruits are known to be used as laxative, poultice, demulcent, emollient, and are also used for food. The fruits are useful in the treatment of several ailments related to the gastrointestinal tract, lungs and bladder. The plant is also reported to be useful in wound infections, ringworm infestations, haemorrhoids, skin diseases and as hypoglycemic, anti-tumour, hypolipidaemic, antiulcer and antifungal treatments [5,6].

Continuing work on the ethnopharmacological wealth of western Himalaya [7], we are working on the validation of the reported therapeutic claims of traditional healers and folk medicine practitioners. Traditionally, the fruit of *F. palmata* L. were prized for its analgesic activity and specifically utilized for backache in the Kumaun region of western Himalaya [7]. So far, there is no study available on the analgesic effect of *F. palmata* and hence this is the first study to establish the same with its possible mechanism of action through docking studies.

## 2. Methodology

### 2.1. Collection and Preparation of Plant Extract

Mature fruits of *F. palmata* were collected from Pithoragarh district of the Uttarakhand state of India, which is situated in the western Himalayan region. The herbarium specimen was prepared and authenticated from Regional Ayurveda Research Institute (RARI), Jhansi and voucher specimen (JHS 25273) was deposited in the herbarium of RARI Jhansi for future reference. Fruits (around 500 g) were then washed, crushed into small pieces and shade dried. Coarse fruit powder (200 g) was then extracted with about 500 mL of hydroethanolic mixture (70% *v*/*v*) for 4 days at room temperature with occasional stirring and then filtered. The extract (200 mL) was then obtained by removing the solvent using a rotary evaporator and coded as FPFE. The yield of the extract was found to be 17.403% *w*/*w*. The extract was further standardized by HPLC along with the marker psoralen which was then confirmed in the extract (Table S1 and Figure S1).

### 2.2. Animals

Wistar rats of either sex weighing 150–200 g were obtained from the animal house of the Department of Pharmaceutical Sciences, Bhimtal Campus, Nainital. The animals were housed in groups of 5 in polypropylene cages (*n* = 6) in a room maintained at a constant temperature (21 °C) and 12 h light: dark cycle, with free access to a standard chow and water ad libitum for the entire duration of the study. All experiments on rats were performed during the light phase of the light: dark cycle. The experimental protocol was approved by the Institutional Animal Ethical Committee of the Department of Pharmaceutical Sciences (IAEC approval No. KUDOPS/27). All possible efforts were made to minimize the suffering of animals and to reduce the number of animals used in the experiments. Group I was the vehicle-treated control, Group II was the positive control diclofenac sodium 10 mg/kg p.o (Dcl), Group III received FPFE 100 mg/kg p.o., Group IV received FPFE 200 mg/kg p.o, and Group V received FPFE 400 mg/kg p.o.

### 2.3. Acute Toxicity Study

Acute toxicity study (ATS) was carried out as per the Organization for Economic Co-operation and Development (OECD) guideline 423 using healthy female Wistar rats which were starved overnight. ATS is used to observe the non-observable adverse effect dose level (NOAEL). Acute toxicity study was carried out for 300 mg/kg dose moving further to 2000 mg/kg dose on rats (*n* = 3) for each dose. Continuous observations in all the rats were made for neurological, behavioral and autonomic profiles for a period of 2 h, 24 h and 72 h and followed by up to 14 days for any moribund state or death.

### 2.4. Hot Plate Test

The test was carried out to assess the effect of the plant extract on thermal nociceptive threshold. Mature albino Wistar rats of either sex (*n* = 6/group) were treated with 100, 200 and 400 mg/kg FPFE, water and 10 mg/kg diclofenac as described in the experimental protocol. The experimental rats were acclimated to the hot plate by placing them for 5 min, 24 h earlier to the test. The reaction times of these rats were measured at 1 h before the treatment, 30, 60, 120 and 240 min after the treatment. The rats were placed in a hot plate analgesiometer (Medicraft heated plate analgesiometer Mark III—Medicraft electro medicals (P) Ltd., Lucknow, India) at 55 °C ± 0.1 and the cut off time of 25 s was set to prevent tissue damage. The latency of rats in licking the hind paw or jumpingwas the pain threshold and was recorded. The rats exhibiting a reaction time over 15 s were not used in the experiment [8,9,10].

### 2.5. Tail Flick Test

Assessment of the tail flicking latency in rats was done by radiant heat analgesiometer (Radiant Heat, Popular traders, Ambala Cantt). Strength of the current was kept constant at 6 amps passed through a naked nichrome wire. Tail skin was placed at a distance 1.5 cm away from the heat source and 10 s cut off reaction time was fixed to prevent tissue damage [11].

### 2.6. Tail Immersion Test

The tail immersion method was used as an important model to evaluate the analgesic potential. The rat’s tail around 4–5 cm from the tip was immersed into a water bath maintained at 55 ± 0.5 °C. The time to flick out the tail was recorded. Similarly to the hot plate test, rats exhibiting a reaction time over 10 s were not used in the experiment [8,9]. The flicking response was calculated as the time taken to withdraw the tail from the hot water source, and the results were compared with the control group.

### 2.7. Formaldehyde Induced Pain Model

The experimental rats were treated with the test drugs and standards as per the protocol described earlier. After one hour of the administration of drugs, 50 μL of 2% formaldehyde solution was injected subcutaneously into the left hind paw of the experimental rats [12]. The spontaneous nociceptive behaviour was immediately determined by measuring the duration of paw licking [13,14,15]. Rats were immediately placed in a transparent plastic cage and the paw licking time and frequency was recorded from initial 0 to 5 min (first-phase, neurogenic) and then 15 to 30 min (second-phase, inflammatory) [16]. The percent (%) inhibition of the duration of licking was also calculated as follows [13]:

Inhibition (%) = [Duration of paw licking (control) − Duration of paw licking (test)] × 100/Duration of paw licking (control).(1)

### 2.8. Statistical Analysis

The results were expressed as mean ± standard error of the mean (SEM). The statistical significance was assessed using one-way analysis of variance (ANOVA) and ANOVA by repeated measured followed by multiple comparison test, i.e., Tukey comparison test, with the help of Graphpad prism software; *p* < 0.05 was considered as significant.

### 2.9. Molecular Docking Studies

*F. palmata* is a poorly explored plant species of *Ficus* genus, and limited literature is available on this plant. A study done by Alqasoumi et al. reported the presence of some prominent phytoconstituents like furanocoumarin psoralen and flavone glycoside rutin [17]. Moreover, we have also identified psoralen in our plant extract by HPLC as mentioned in Section 2.1. Therefore, taking into consideration the therapeutic importance of these phytoconstituents, we hypothesized that the potent analgesic activity of the plant extract might be due to the interaction of either one or both of these constituents via COX enzyme inhibition or through mu-opioid receptors. Molecular docking studies were performed against COX-2 (PDB ID: 3NT1) and mu-opioid (PDB ID: 4DKL) receptors using diclofenac (as a standard molecule), psoralen and rutin.

All the structures were collected from the PubChem database. These were prepared in Chimera as well as in AutoDock tools before docking in AutoDock Vina. The grid box centre was calculated using co-crystallized ligand in PDB. The grid box was prepared around the active site with dimension 25 × 25 × 25 and spacing 0.375 [18]. First co-crystallized ligand (Naproxan bound in PDB: 3NT1 and Morphinan bound in PDB: 4DKL) of each PDB file was docked into the active site. This was done to validate the AutoDock Vina program, to see whether it was able to generate the same binding interactions as those present in PDB or not. Then, using the same parameters, diclofenac, psolarlen and rutin were also docked into the active site of PDB IDs 3NT1 and 4DKL. The results of these studies were analysed for binding affinity and interactions.

## 3. Results and Discussion

### 3.1. Chemical Characterization of Plant Extract

Under optimized conditions of chromatography, the retention time (Rt) of psoralen was 9.695 min. The specificity of the method was established by comparison the HPLC retention times and absorption spectra peaks from the analyzed samples with the reference standard. The sensitivity of the method were examined by the limit of detection (LOD) and limit of quantification (LOQ) which were found to be 5.0 ng/mL and 20.0 ng/mL, respectively. The HPLC results clearly revealed the presence of psoralen in the hydroalcoholic extract of *F. palmata.* We have also reported a detailed analysis of the extracts of *F. palmata* by HPTLC and UPLC-MS [19,20] and found that multiple compounds like elagic acid, psoralen, gallic acid aliphatic and aromatic carboxylic acids, along with many flavonoids, were found in the *F. palmata* extracts.

### 3.2. Acute Toxicity Study

No sign of moribund state or toxicity was recorded in animals that received 2000 mg/kg FPFE. Therefore, LD_50_ of FPFE was considered over 2000 mg/kg according to GHS classification (OECD, 2001). As, median effective dose (ED_50_) is one-tenth of the LD_50_, hence, 200 mg/kg turned out to be the ED_50_ of FPFE, and 200 mg/kg and 400 mg/kg doses were selected for the study.

### 3.3. Hot Plate Test

**Figure 1:**
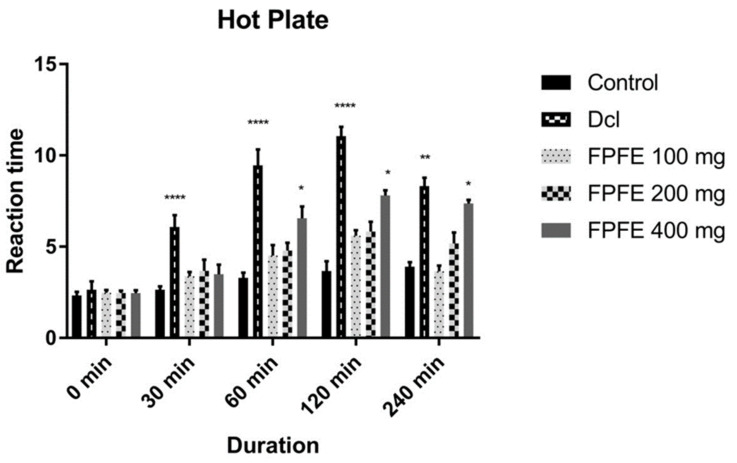
Effect of different doses of FPFE on the latency in reaction time of rats exposed to hot plate (* *p* < 0.05; ** *p* < 0.01; **** *p* < 0.0001).

The analgesic effect of *F. palmata* fruit extract on the experimental rats as evaluated by Eddy’s hot plate method is shown in Figure 1. The results revealed that the diclofenac-treated group showed a significant (*p* < 0.0001) increase in reaction time compared to control and other groups up to 240 min after its administration. The 100 mg/kg, 200 mg/kg and 400 mg/kg doses of FPFE also prolonged the latency period of paw licking and jumping response in rats as compared to the control group, and marked difference in the prolongation of latency period was recorded from 400 mg/kg FPFE after 60 min duration. The highest effect in prolonging the latency period was recorded from the FPFE 400 mg/kg dose. The extract showed a time-dependent effect on the latency period. After 240 min of the administration, the FPFE 400 mg/kg dose exhibited a significant (*p* < 0.05) effect in reaction time as compared to the control. Although the effect was significant, however, it was not higher than that of the standard drug. The results revealed that FPFE showed a significant analgesic activity when evaluated with Eddy’s hot plate method. The effect was also dose dependent and time dependent.

### 3.4. Tail Flick Test

**Figure 2:**
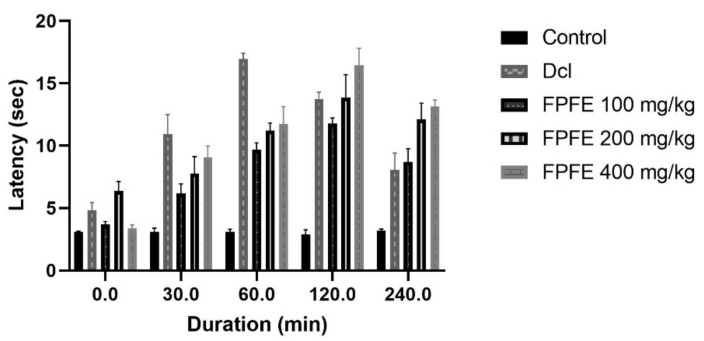
Effect of different doses of FPFE on the reaction time (s) in rat’s tail flick test.

Tail flick test by latent heat confirmed the analgesic effect of FPFE at different doses. The dose of 400 mg/kg of FPFE showed a comparable effect to diclofenac after 30 min; after 60 min duration, the effect was decreased. Higher and significant (*p* < 0.01) effect was observed from the 200 mg/kg dose after the period increased. FPFE at 100 mg/kg and 400 mg/kg dose revealed highest significant (*p* < 0.001) effect after 120 min as compared to control. Subsequently, after 240 min, 400 mg/kg of FPFE again exhibited significant (*p* < 0.001) analgesic effect followed by 200 mg/kg FPFE, which also showed significant (*p* < 0.01) effect on the reaction time as presented in Figure 2. In this model, the effect of the tested drug was found higher than the standard drug, revealing the higher potency of the plant extract. However based upon only one test, it cannot be concluded that the extract displayed better activity than the standard. The difference in the 200 and 400 mg/kg dose of FPFE showed that the drug displayed good activity on the higher dose.

### 3.5. Tail Immersion Test

**Figure 3:**
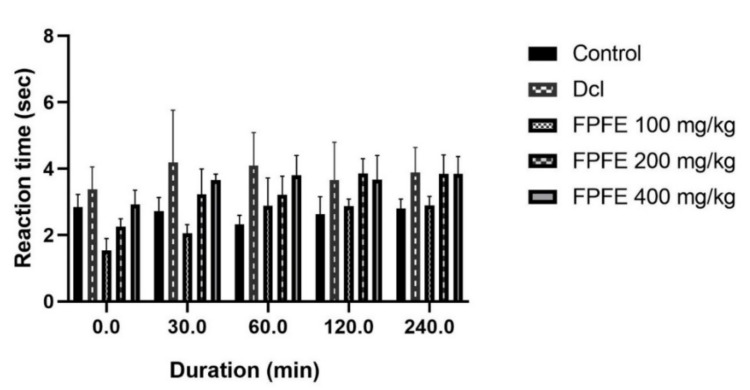
Effect of different doses of FPFE on the reaction time in rat’s tail immersion test.

The tail immersion method also showed the analgesic effect from the FPFE at different doses. The results of the test varied from the results obtained from the hot plate test. This test revealed that 200 mg/kg and 400 mg/kg doses of the FPFE exhibited an increase in the reaction time as compared to the control group. However, 100 mg/kg dose of FPFE initially showed less reaction time than control, however, after 60 min it showed a prominent analgesic effect. The 200 mg/kg dose of FPFE revealed significant analgesic activity after 120 (*p* < 0.01) and 240 (*p* < 0.05) minutes. Additionally, 400 mg/kg FPFE dose also showed a similar and equivalent effect to that of 200 mg/kg dose after 240 min. The results are presented in Figure 3. The analgesic effect of FPFE was comparable to the standard analgesic drug diclofenac, but it was not more than the standard, as also recorded from the hot plate test.

### 3.6. Formaldehyde Induced Pain Model

**Figure 4:**
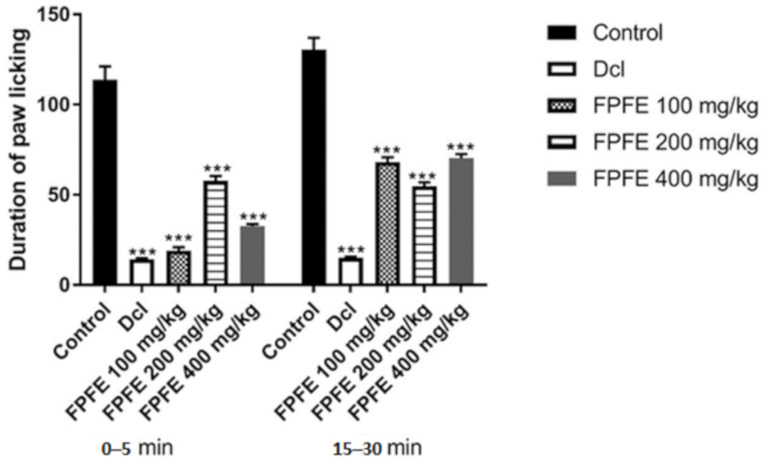
Effect of FPFE at all the tested doses in both early neurogenic and late inflammatory phases estimated by formalin induced pain models. FPFE = F. palmata fruit extract; Dcl = diclofenac, duration of licking is presented as mean ± SEM (*n* = 6). *** *p* < 0.001 versus vehicle-treated control using one-way ANOVA followed by Tukey’s post hoc multiple-comparison test.

FPFE treatment significantly (*p* < 0.05) reduced the paw licking tendency in rats of the paw injected with formalin in the early and late phase of the treatment when compared to control. Licking frequency was significantly (*p* < 0.05) reduced in the early as well as late phase, but it was more apparent during the early phase. FPFE at all the tested doses showed a significant effect (*p* < 0.05) in both early neurogenic and late inflammatory phases (Figure 4).

In the early phase (0–5 min) the duration of licking was recorded as 113.66 ± 7.48 s, and for the late phase (15–30 min) it was 130.5 ± 6.53 s in the control group. Diclofenac produced a significant decline in the duration of paw licking activity in both phases. It showed a % inhibition of 87.9% and 88.5% in the early and late phases, respectively. A marked reduction was recorded from the 100 mg/kg dose of FPFE in the early phase, which exhibited 83.76% inhibition. The effect was not found to be dose dependent as 200 and 400 mg/kg FPFE showed 48.53% and 71.41% inhibition, respectively. In the late phase, the % inhibition was found comparatively low when compared to the early phase of the respective doses. Doses of 100 mg/kg showed 47.77%, 200 mg/kg 57.98% and 400 mg/kg dose showed 46.10% inhibition.

### 3.7. Docking Studies against COX-2 (PDB: 3NT1)

**Figure 5:**
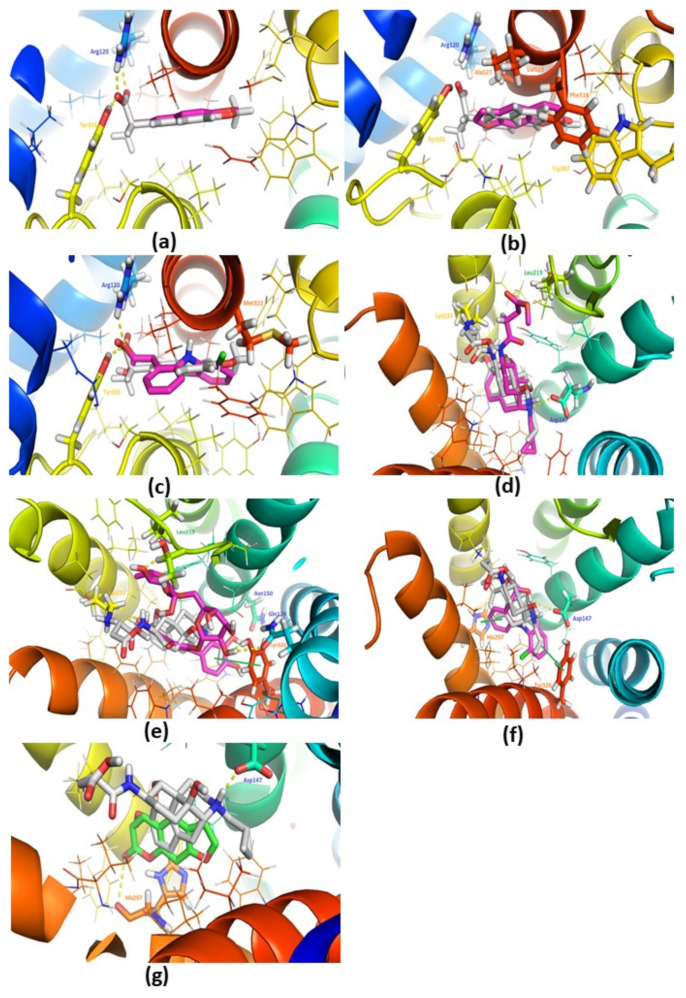
(**a**) Docking pose of X-ray ligand (Naproxen) into the active site of 3NT1, and Magenta (docked) colour sticks represent Naproxen, Blue and yellow sticks represent residues involved in H-bonding interactions. (**b**) Docking pose of Psoralen into the active site of 3NT1, White (X-ray: Naproxen) and Magenta (docked) colour sticks represent Psoralen. Other sticks and lines represent active site residue. (**c**) Docking pose of diclofenac into the active site of 3NT1, White (X-ray: Naproxen) and Magenta (docked) colour sticks represent diclofenac, Blue and yellow sticks represent residues involved in H-bonding interactions. (**d**) Docking pose of Morphinan Antagonist (X-ray) into the active site of 4DKL, White (X-ray) and Magenta (docked) colour sticks represent Morphinan Antagonist, Cyan, yellow and green sticks represent residues involved in H-bonding interactions. (**e**) Docking pose of Rutin into the active site of 4DKL, White sticks for X-ray: Morphinan Antagonist and Magenta (docked) sticks for Rutin, Orange, yellow, green and Cyan sticks represent residues involved in H-bonding interactions. (**f**) Docking pose of diclofenac into the active site of 4DKL, White (X-ray: Morphinan Antagonist) and Magenta (docked) colour sticks represent diclofenac, Cyan sticks represent residues involved in H-bonding interactions. (**g**) Docking pose of Psoralen into the active site of 4DKL, White sticks for X-ray: Morphinan Antagonist and Magenta (docked) sticks for Psoralen, Orange and Cyan sticks represent residues involved in H-bonding interactions. White sticks are for X-ray ligand in case of PDB: 3NT1 in (**a**–**c**) and White sticks for X-ray: Morphinan Antagonist in (**g**), Yellow dots represent H-bonding interactions, Dark green lines represent pi-pi interactions. H-bonding and pi-pi interactions are also evaluated by plip program.

The docking studies were performed against active site of COX-2 (PDB IDL 3NT1) using naproxen (co-crystalized ligand of 3NT1), diclofenac (as a standard in in vivo studies), psoralen and rutin. The docking results of naproxen are illustrated in Figure 5a. In docking studies, the naproxen is bound similarly as reported in PDB (almost overlapped with the co-crystallized ligand Figure 5a). On the other hand, the psoralen (Figure 5b, Table 1) exhibited good binding interactions and score as compared to diclofenac (Figure 5c, Table 1).

However, psoralen is exhibited low binding score as compared to naproxen which is marketed drug against COX-2 (Figure 5b). The rutin is exhibited the lower binding score against 3NT1 (Table 1). Besides, psoralen is also sharing the common interactions (H-Bonding with Arg120 and Van der Waals: Leu352, Trp387, Val523) as found with the standard drugs (naproxen and diclofenac). Therefore, psoralen is found to have high binding potential as compared to Diclofenac against COX-2 (Table 1). These results are supported by the published data [21]. The results of these studies suggested that psoralen could be a potential therapeutic candidate against COX-2 as an anti-inflammatory and analgesic drug, but need to tested in in vivo studies separately.

### 3.8. Docking Studies with the Mu-Opioid (PDB: 4DKL)

The docking studies were performed against active site of mu-opioid (PDB: 4DKL) using morphinan antagonist (co-crystalized ligand of 3NT1), diclofenac (as a standard drug in in vivo studies), psoralen and rutin. The docking results of morphinan antagonist are illustrated in Figure 5d. In docking studies, the morphinan antagonist is also bound similarly as found in PDB (overlapped with co-crystalized ligand Figure 5d). It was found that rutin Figure 5e; (H-bonding with Gln124, Asn150, Leu219, Lys233, Tyr326) had the highest binding score as compared to the morphinan antagonist (H-Bonding with Asp147, Leu219, Lys233) and diclofenac Figure 5f; (H-Bonding with Asp147 only). Due to extra interactions (H-bonding: Gln124, Asn150, Tyr326, π-Stacking: Tyr326) with rutin (Figure 5e), a high binding score was exhibited, as compared to the morphinan antagonist. However, psoralen (Figure 5g) had almost the same binding affinity as compared to diclofenac, but lower than rutin (Table 1). Moreover, psoralen and rutin shared the common Van der Waals interactions (Ile296, Val300) as also found with the morphinan antagonist and diclofenac (Table 1). The results of these studies show that rutin possesses high binding potential (high binding score and interactions). This could be a potential therapeutic candidate against mu-opioid, but needs to be tested in in vivo studies.

## 4. Discussion

There is an emerging interest in the use of medicinal plants in the treatment of numerous disease conditions. Ethnopharmacological information is of prime importance for the discovery of potential therapeutically important molecules which are either obtained directly from natural sources or inspired by nature [6]. *F. palmata* is an important plant of the western Himalayan region in terms of its medicinal potential. We have conducted detailed chemical characterization of *F. palmata* and found that the fruit extract contains rutin and psoralen along with numerous phytoconstituents. We have also found that the fruits of the plant possess good antioxidant and enzyme inhibitory activities [19]. The analgesic activity of FPFE was evaluated by well-accepted models like the hot plate, tail-flick, tail immersion and formalin tests.

These results revealed that FPFE simultaneously possesses analgesic activity on both central and peripheral pain pathways. A similar mechanism was reported for the analgesic effect of *Polygonum orientale* L. [13]. NMDA receptors in the spinal cord are also known to a play a role in hyperalgesia developed due to intraplantar injection of formalin, and the inhibitory effect on hyperalgesia is believed to be due to the involvement of neuronal inhibition by GABA receptor activation [14,22,23]. The results also indicate that the antinociceptive effect of FPFE may be mediated by glutamate mediators and inflammatory mediators like prostaglandins, excitatory amino acids and histamine.

Tests involving thermal stimuli for the evaluation of nociception and pain have been discussed over a substantial period of time [24]. The tail-flick model in rodents [25] is one of the standard methods for the investigation of nociception and analgesia [26]. Both hot plate and tail-flick are thermal models of pain, however, the tail-flick model generally refers to a spinal reflex with modest control through supraspinal structures; it is a well-established model for the evaluation of opioid derived analgesics [27]. The hot plate model is believed to be more complex than the tail-flick model, due to paw licking and jumping responses, which are the two important behavioral components and are considered to be supraspinally integrated [28,29]. Hot plate is a commonly used model to evaluate the analgesic and centrally-mediated anti-nociceptive effects. The inflammatory pain is promoted by cyclooxygenase pathway via conversion of arachidonic acid to PGE2 by means of COX-2, which is an important inflammatory mediator [30]. In this study FPFE significantly increased the latency of response to painful stimulus which is suggestive of its anti-nociceptive effect. The formaldehyde-induced pain model is a response of biphasic nature. In the neurogenic (first 0 to 5 s) phase of pain, there is the involvement of glutamate mediators of formaldehyde-induced pain [31]. Inflammatory mediators like prostaglandins, excitatory amino acids and histamine come to play in the second phase (15 to 30 min) of the inflammatory pain response. Interestingly, bradykinin shows a unique property of affecting both phases at the same time [13,32]. Our findings indicate that FPFE at different doses demonstrated the potential effect on pain suppression by modifying the analgesic effect of formaldehyde in both phases.

In addition, molecular docking studies were also performed to evaluate the role of *F. palmata* phytochemicals in pain management using COX-2 and mu-opioid receptors. These studies suggested that psoralen has high binding affinity and interactions against COX-2 (as compared to the standard drug: diclofenac) and rutin has high binding affinity and interactions (as compared to morphinan antagonist (co-crystalized ligand in PDB: 4DKL) and diclofenac. The results are in line with the results obtained through in vivo studies. Limited in vitro studies have been performed for psoralen against COX-2 and mu-opiod receptors. In an earlier study, psoralen isolated from *Dystaenia takeshimana* was reported to inhibit COX-2/5-LOX in mouse bone marrow-derived mast cells [33]. A few studies suggest that psoralen derivatives [34,35] and rutin [36,37,38] possess COX-2 inhibitory activity. For instance, 5-methoxy-8-(2-hydroxy-3-buthoxy-3-methylbutyloxy)-psoralen from *Angelica dahurica* was reported to inhibit induction of COX-2 in bone marrow-derived mast cells [34]. Several reports are also available exhibiting that rutin [39,40,41,42] were found to have anti-opioid activity. The previous reports also support our data, as they show that psoralen has high binding potential against COX-2 receptor (PDB: 3NT1) and low potential with mu-opioid (PDB: 4DKL) receptor.

The results revealed that the hydroalcoholic extract of *F. palmata* fruits has analgesic potential. Further, the findings also demonstrated that this effect was due to different inflammatory mediators, modifications in their pathways and other centrally acting mechanisms. Further scientific studies are required to evaluate the molecular mechanism of the analgesic activity and effect of *F. palmata* fruits on different inflammatory mediators like PGE2, TNF-α, IL-6, and IL-1β. In addition, a comprehensive metabolomic characterization of *F. palmata* is required to understand the phytochemical diversity and further identification of the compound responsible for the analgesic activity. Our study supported the use of *F. palmata* fruits in different analgesic conditions like joint pain by the inhabitants of rural areas of the western Himalayan region of Uttarakhand. The molecular docking studies of phytochemicals (psoralen and rutin) of *F. palmata* were also performed against analgesic targets (like COX-2 and mu-opioid receptors) to understand the mechanism action for analgesic effects of *F. palmata*. It is shown that psoralen has high binding potential against COX-2 receptors (PDB: 3NT1), but low potential with mu-opioid (PDB: 4DKL) receptors. Additionally, rutin has high binding potential against mu-opioid receptors.

## 5. Conclusions

In summary, FPFE showed potential analgesic effects at different doses in general and at a 400 mg/kg dose in particular when observed in the analgesic models of central and inflammatory pain. The results also revealed that the phytoconstituents present in *F. palmata* showed their effects through COX-2 and mu-opioid receptors. These results demonstrate that the fruit of *F. palmata* have a potential analgesic effect, and its traditional consumption also makes it a safe alternative for pain management in the rural areas of Uttarakhand. Therefore, these studies provide a pharmacological basis for the traditional use of *F. palmata* fruits in pain management. In addition, limited in vitro studies have been performed to understand the comprehensive molecular mechanism of psoralen and rutin in pain management. Hence, such studies will be beneficial to understand the mechanistic insights of various specialized metabolites against pain management in future.

## Figures and Tables

**Table 1:** Molecular docking results against COX-2 (3NT1) and mu opioid (4DKL) receptors.

Mole	PDB ID	Score (Kcal/mol)	Interactions		
			H-Bonding	Van der Waals	Other
Naproxen (X-Ray)	3NT1	−9.20	**Arg120**, Tyr355	Val394, Leu352, Tyr355, Leu359, Val523, Ala527, Leu531	Salt Bridge: Arg120
Diclofenac	3NT1	−7.20	**Arg120**, Tyr355, Val523	Val349, Leu352, Tyr385, Trp387, Val523, Ala527	Halogen bond: Met522 Salt Bridge: Arg120
**Psoralen**	**3NT1**	**−8.40**	**Arg120**	**Leu352, Trp387, Val523**	
Rutin	3NT1	−6.80	Arg120, Tyr355, Met522, Val523, Glu524, Ser530	Val89, Leu93, Trp100, Ile112, Val116, Val349, Leu531	π-cation: Arg120 Salt Bridge: Arg120
Morphinan Antagonist (X-ray)	4DKL	−9.00	Asp147, Leu219, Lys233	Leu219, Trp293, Ile296, Val300, Ile322, Tyr326,	Salt Bridge: Asp147
**Diclofenac**	**4DKL**	**−7.40**	**Asp147**	**Trp293, Ile296, Val300**	**π-Stacking: His297, Tyr326**
**Psoralen**	**4DKL**	**−7.20**	**His297**	**Val236, Ile296, Val300**	**Salt Bridge: His297**
Rutin	4DKL	−9.60	Gln124, Asn150, Leu219, Lys233, Tyr326	Lys233, Ile296, Val300, Ile322	π-Stacking: Tyr326

## Data Availability

Not applicable.

## Supplementary Materials

The following are available online at https://www.mdpi.com/article/10.3390/plants10081685/s1, Figure S1: RP-HPLC chromatogram of psoralen standard (A) and psoralen identified in *F. palmata* sample (B). Table S1: Gradient conditions of the mobile phase for HPLC.

## Abbreviations

FPFE	*Ficus palmata* L. fruit extract
NSAIDs	non-steroidal anti-inflammatory drugs
RARI	Regional Ayurveda Research Institute
IAEC	Committee of the Department of Pharmaceutical Sciences
ATS	Acute toxicity study
OECD	Organization for Economic Co-operation and Development
NOAEL	non-observable adverse effect dose level
SEM	standard error of the mean
HPLC	High-performance liquid chromatography
ANOVA	Analysis of variance
LOD	limit of detection
LOQ	limit of quantification
UPLC-MS	Ultra performance liquid chromatography-mass spectrometry
PDB	The Protein Data Bank
