# Supplementary material for: Himalayan Ficus palmata L. Fruit Extract Showed In Vivo Central and Peripheral Analgesic Activity Involving COX-2 and Mu Opioid Receptors
Source: Plants (Basel). 2021 Aug 16;10(8):1685. doi: 10.3390/plants10081685 (PMC8398767; doi:10.3390/plants10081685)
Supplement: Supplementary file 1 [file plants-10-01685-s001.zip › plants-1260712-supplementary.pdf]

plants

Supplementary material

Himalayan *Ficus palmata* L. Fruit Extract Showed In Vivo Central and Peripheral Analgesic Activity Involving COX-2 and Mu Opioid Receptors

Table S1. Gradient conditions of the mobile phase for HPLC.

Time	Flow Rate	% of solvent A	% of Solvent B
0.01	1.5	75	25
18.00	1.5	50	50
25.01	1.5	25	75
32.0	1.5	20	80
35.0	1.5	50	50
40.0	1.5	75	25
45.0	1.5	75	Stop

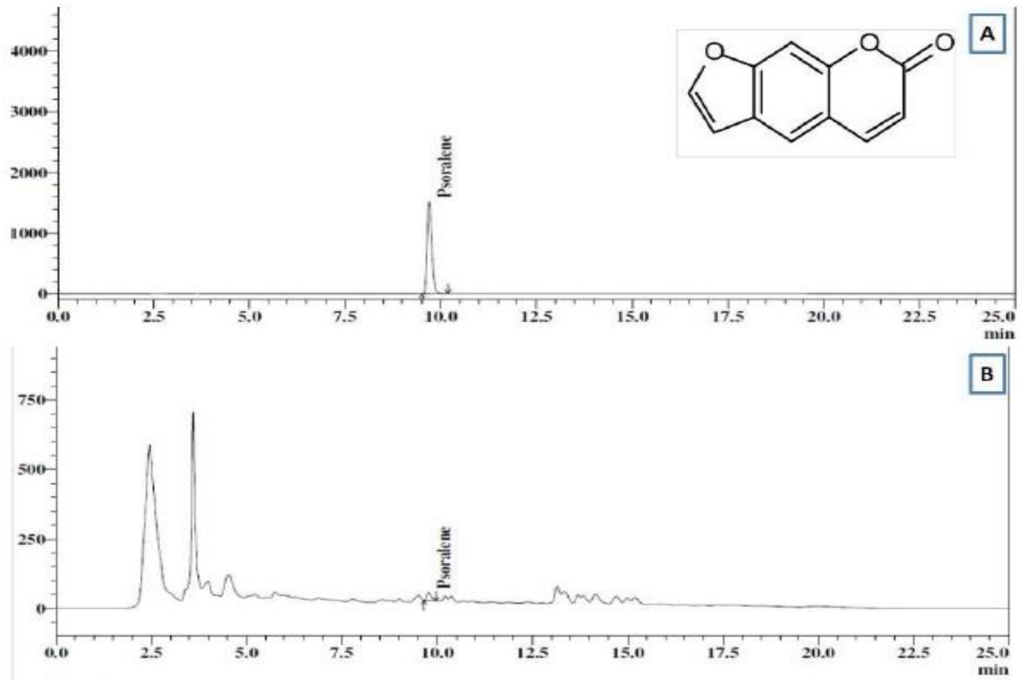

Figure S1. RP-HPLC chromatogram of psoralen standard (**A**) and psoralen identified in *F. palmata* sample (**B**).
